# FOXO3, Telomere Dynamics, and Healthy Brain Aging: A COBRE Study

**DOI:** 10.1093/geroni/igab046.1419

**Published:** 2021-12-17

**Authors:** Kalpana Kallianpur, Bradley Willcox, Kamal Masaki, Richard Allsopp

**Affiliations:** 1 Kuakini Medical Center, Honolulu, Hawaii, United States; 2 Kuakini Medical Center, Kuakini Medical Center/Honolulu, Hawaii, United States

## Abstract

Human longevity is linked to genetic, cellular, and other complex biological and psychosocial traits. Aging is typically accompanied by gradual brain atrophy and cognitive decline, but the mechanisms are unclear. Cellular aging, characterized by telomere shortening and altered telomerase activity, is related to mortality and brain aging. Decelerated brain aging is associated with greater peripheral blood leukocyte telomere length (LTL) and, we hypothesize, may be linked to FOXO3 genotype. We will use MRI to assess brain structure and function cross-sectionally in 100 Kuakini Honolulu Heart Program Offspring. Atrophy and disrupted functional connectivity, markers of brain aging, will be examined in relation to FOXO3 and LTL. Associations between brain structural and functional differences, FOXO3 genotype and LTL will be investigated over a wide range of ages, controlling for other biological and psychosocial factors. Results may provide insight into mechanisms influencing the rate of brain aging, and may eventually extend human healthspan.

